# Draft genome sequence of *Francisella tularensis subsp. holarctica* BD11-00177

**DOI:** 10.4056/sigs.4217923

**Published:** 2013-08-10

**Authors:** Jordy P. M. Coolen, Andreas Sjödin, Boulos Maraha, Gerard F. Hajer, Mats Forsman, Ellen Verspui, Hendrina M.E. Frenay, Daan W. Notermans, Maaike C. de Vries, Frans A.G. Reubsaet, Armand Paauw, Guus Roeselers

**Affiliations:** 1 TNO, The Netherlands; 2 Division for CBRN Defence and Security, FOI - Swedish Defence Research Agency, Umeå, Sweden; 3 Department of Medical Microbiology, Beatrix Hospital, Gorinchem and Albert Schweitzer Hospital, Dordrecht, The Netherlands.; 4 Department of Surgery, Beatrix Hospital, Gorinchem, The Netherlands; 5 Public Health Service Zuid Holland Zuid, Dordrecht, The Netherlands; 6 Diagnostic Laboratory for Infectious Diseases and Perinatal Screening (LIS), Center for Infectious Disease Control, National Institute of Public Health and the Environment (RIVM), Bilthoven, The Netherlands

**Keywords:** tularaemia, biodefence, zoonotic infection, phylogeography, Netherlands

## Abstract

*Francisella tularensis* is a facultative intracellular bacterium in the class *Gammaproteobacteria*. This strain is of interest because it is the etiologic agent of tularemia and a highly virulent category A biothreat agent. Here we describe the draft genome sequence and annotation of *Francisella tularensis subsp. holarctica* BD11-00177, isolated from the first case of indigenous tularemia detected in The Netherlands since 1953. Whole genome DNA sequence analysis assigned this isolate to the genomic group B.FTNF002–00, which previously has been exclusively reported from Spain, France, Italy, Switzerland and Germany. Automatic annotation of the 1,813,372 bp draft genome revealed 2,103 protein-coding and 46 RNA genes.

## Introduction

*Francisella tularensis* is a Gram negative, non-motile, non-spore forming, facultative intracellular bacterium appearing as short rods or coccoid forms [1]. *F. tularensis* is the etiologic agent of tularemia, a zoonotic infection also known as rabbit fever and deer-fly fever. Transmission to humans has been reported by direct contact with infected animals, arthropod bites, inhalation of contaminated dust or ingestion of contaminated food or water. This pathogen is highly infectious as it can cause infection upon inhalation of as few as 10 cells. This extremely low infectious dose makes transmission via aerosols easy, and previous attempts to weaponize this microorganism have led to its recognition as a category A biothreat agent (CDC classification) [2,3]. *F. tularensis* contains three subspecies that are infectious to humans; the highly virulent *Francisella tularensis subsp. tularensis*, which often causes a lethal multi-systemic disease with a fatality rate of up to 30%, the less virulent *Francisella tularensis* subsp. *holartica* and *Francisella tularensis subsp. mediasiatica*, which both seldom cause infectious in humans. Here we present a summary classification together with the description of the draft genome sequence and annotation of *Francisella tularensis subsp. holarctica* BD11-00177, that was isolated from a vesicle on the forehead of a 72-year-old male living in The Netherlands. As the patient had not been abroad for years, this was the first documented case of indigenous tularemia in The Netherlands since 1953.

## Classification and features

**Figure 1:**
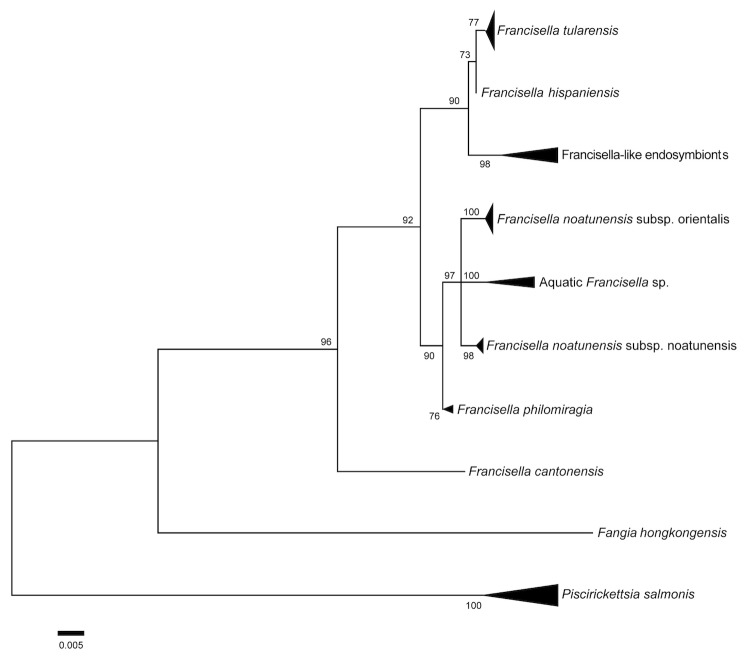
Maximum likelihood tree illustrating the phylogenetic relationships among several members of the genus *Francisella* and members of the order *Thiotrichales* based on full-length 16S rRNA gene sequences.

*Francisella* is the only genus within the family *Francisellaceae* and is a member of the order *Thiotrichales* and the class *Gammaproteobacteria* [4] [Table 1]. Besides *F. tularensis*, the genus *Francisella* includes the species *Francisella halioticida, Francisella hispaniensis, Francisella noatunensis*, *Francisella novicida, Francisella philomiragia, Francisella cantonensis* and the misclassified *Wolbachia persica* [4,17, Figure 1]. Only rare human infections with *F. hispaniensis* and *F. novicida*, and *F. philomiragia* are described, often caused after nearly drowning [18,19]. *F. tularensis* is capable of infecting hundreds of different vertebrate and invertebrate hosts [20]. The most widely distributed subspecies is *F. tularensis subsp. holarctica*, which is found throughout much of the Northern Hemisphere and is the only subspecies naturally occurring in Europe [21].

**Table 1:** Classification and general features

**MIGS ID**	**Property**	**Term**	**Evidence code**^a^
		Domain *Bacteria*	TAS [5]
		Phylum *Proteobacteria*	TAS [6]
		Class *Gammaproteobacteria*	TAS [7,8]
	Current classification	Order *Thiotrichales*	TAS [7,9]
		Family *Francisellaceae*	TAS [7-10]
		Genus *Francisella*	TAS [11-14]
		Species *Francisella tularensis*	TAS [11,12]
		Subspecies *Francisella tularensis holarctica*	TAS [15,16]
		Strain BD11-00177	NAS
	Gram stain	negative	TAS [1].
	Cell shape	short rods or coccoid forms	TAS [1].
	Motility	No	TAS [1].
	Sporulation	No	TAS [1].
	Temperature range	Mesophilic	TAS [1].
	Optimum temperature	37	IDA
	Carbon source	Carbohydrates	TAS [1].
	Energy source	Chemoorganotrophic	TAS [1].
	Terminal electron receptor	Facultative anaerobe	TAS [1].
MIGS-6	Habitat	Host	TAS [1].
MIGS-15	Biotic relationship	Obligate host-dependent	TAS [1].
MIGS-16	Host name Host taxon ID Host gender	Homo sapiens 9606 Male	TAS [1]. TAS [1]. NAS
MIGS-14	Pathogenicity Biosafety Level	Pathogen 3	TAS [2].
MIGS-4	Geographic location	The Netherlands	IDA
MIGS-5	Sample collection time	October 2011	IDA
MIGS-4.1	Latitude	unknown	
MIGS-4.2	Longitude	unknown	
MIGS-4.3	Depth	unknown	
MIGS-4.4	Altitude	unknown	
MIGS-4.5	Isolation site Isolation source	Human host vesicle on the forehead	IDA IDA

^a^Evidence codes - IDA: Inferred from Direct Assay; TAS: Traceable Author Statement [i.e., a direct report exists in the literature); NAS: Non-traceable Author Statement [i.e., not directly observed for the living, isolated sample, but based on a generally accepted property for the species, or anecdotal evidence). These evidence codes are from the Gene Ontology project.

## Genome sequencing information

### Genome project history

Strain BD11-00177 was sequenced because of its relevance to biodefense. The draft genome sequence was finished in August 2012. The GenBank accession number for the project is 177784. The genome project is listed in the Genome OnLine Database (GOLD) [22] as project Gi21611. Sequencing was carried out at the Dutch Organization for Applied Scientific Research (TNO) and the Swedish Defense Research Agency (FOI). Initial automatic annotation was performed using the DOE-JGI Microbial Annotation Pipeline (DOE-JGI MAP). Table 2 shows the project information and its association with MIGS 2.0 compliance.

**Table 2:** Project information

MIGS ID	Property	Term
MIGS-31	Finishing quality	Standard Draft
MIGS-29	Sequencing platforms	Illumina MiSeq, 454 Roche GS Junior
MIGS-31.2	Fold coverage	713×
MIGS-30	Assemblers	Ray Assembler V2.1
MIGS-32	Gene calling method	Prodigal [23]
	GOLD ID IMG Taxon ID NCBI PROJECT ID	Gi21611 1244086 177784
MIGS-38	Project relevance	Medical, biodefence

### Growth conditions and DNA isolation

For DNA preparation, strain BD11-00177 was grown on 5% sheep blood agar plates for 72 h at 35°C in the presence of 5% CO_2_. DNA was extracted using the Qiamp DNA Micro Kit according manufacturers guidelines (Qiagen, Westburg b.v., Leusden, The Netherlands).

### Genome sequencing and assembly

Sequencing was performed by the Microbiology and Systems Biology group at TNO and the Division for CBRN Defence and Security at FOI using 454 Roche GS Junior and the Illumina MiSeq platforms. The initial draft assembly yielded 95 large (>1,000 bp) and 86 small (<1,000 bp), non-redundant contigs of 1,813,372 bp by combing 75,245 Roche/454 reads at 23× coverage and 8,289,332 Illumina reads at 690× coverage by hybrid assembly through the Ray Assembler V2.1 [24].

### Genome annotation

Open Reading Frames (ORFs) were predicted using the Prodigal gene prediction algorithm [23] as part of the DOE-JGI Microbial Annotation Pipeline (DOE-JGI MAP) using default parameters, followed by a round of manual curation. CRISPR elements were predicted using CRT and PILERCR [25]. Predictions from both methods were concatenated. Identification of tRNAs was performed using tRNAScan. Ribosomal RNA genes (5S, 16S, 23S) are predicted using the program RNAmmer [26]. With the exception of tRNA and rRNA, all models from Rfam [27] are used to search the genome sequence. For faster detection, sequences are first compared to a database containing all the ncRNA genes in the Rfam database using BLAST, with a very loose cutoff. Subsequently, sequences that have hits to any genes belonging to an Rfam model are searched using the program INFERNAL [27]. Protein coding genes were compared to protein families (e.g., COGs, Pfam, KEGG) and the proteome of selected “core” genomes, which are publicly available, and the product names were assigned based on the results of these comparisons.

## Genome properties

The genome was assembled into 95 large (>1,000 bp) contigs and includes one circular chromosome with a total size of 11,813,372 bp (32.23% GC content). A total of 2,149 genes were predicted, 2,103 of which are protein-coding genes. Of the protein coding genes, 1,592 were assigned to a putative function, with the remaining being annotated as hypothetical proteins. The properties and the statistics of the genome are summarized in Tables 3 and 4.

**Table 3:** Nucleotide content and gene count levels of the genome

**Attribute**	Value	% of total^a^
Genome Size (bp)	1,813,372	100.00%
DNA coding region (bp)	1,611,603	88.87%
DNA G+C content (bp)	584,435	32.23%
Total genes^b^	2149	100.00%
RNA genes	46	2.14%
Protein-coding genes	2103	97.86%
Genes in paralog clusters	1262	58.72%
Genes assigned to COGs	1584	73.71%
Protein coding genes connected to KEGG pathways	611	28.43%
not connected to KEGG pathways	1492	69.43%
Genes with signal peptides	111	5.17%
Genes with transmembrane helices	573	26.66%

a) The total is based either on the size of the genome in base pairs or on the total number of protein coding genes in the annotated genome.

**Table 4:** Number of genes associated with the 25 general COG functional categories

**Code**	**Value**	**%age**^a^	**Description**
J	152	8.79	Translation
A	1	0.06	RNA processing and modification
K	65	3.76	Transcription
L	198	11.45	Replication, recombination and repair
B	-	-	Chromatin structure and dynamics
D	18	1.04	Cell cycle control, mitosis and meiosis
Y	-	-	Nuclear structure
V	31	1.79	Defense mechanisms
T	24	1.39	Signal transduction mechanisms
M	112	6.47	Cell wall/membrane biogenesis
N	19	1.1	Cell motility
Z	1	0.06	Cytoskeleton
W	-	-	Extracellular structures
U	44	2.54	Intracellular trafficking and secretion
O	66	3.82	Posttranslational modification, protein turnover, chaperones
C	107	6.18	Energy production and conversion
G	118	6.82	Carbohydrate transport and metabolism
E	158	9.13	Amino acid transport and metabolism
F	65	3.76	Nucleotide transport and metabolism
H	96	5.55	Coenzyme transport and metabolism
I	64	3.7	Lipid transport and metabolism
P	71	4.1	Inorganic ion transport and metabolism
Q	37	2.14	Secondary metabolites biosynthesis, transport and catabolism
R	172	9.94	General function prediction only
S	111	6.42	Function unknown
-	565	26.29	Not in COGs

a) The total is based on the total number of protein coding genes in the annotated genome.

## Comparisons with other fully sequenced genomes

Comparison of the assembled draft genome sequence of strain BD11-00177 with publicly available *F. tularensis* genome sequences revealed that it clusters in the FTNF002-00 genomic group (B.Br.FTNF002-00 and BIV.FTNF002-00) defined by the FTNF002-00 genome sequence [28-30] within the B.IV clade. The presence of the 1.59 kb RD23 deletion event [31] as well as the 464 bp size of the MLVA marker FtM24 [32], both typical for the FTNF002-00 genomic group, were confirmed *in silico*. Notably, isolates from this genomic group had previously been exclusively reported from Spain, France, Italy, Switzerland and Germany [28,31-35].

**Figure 2:**
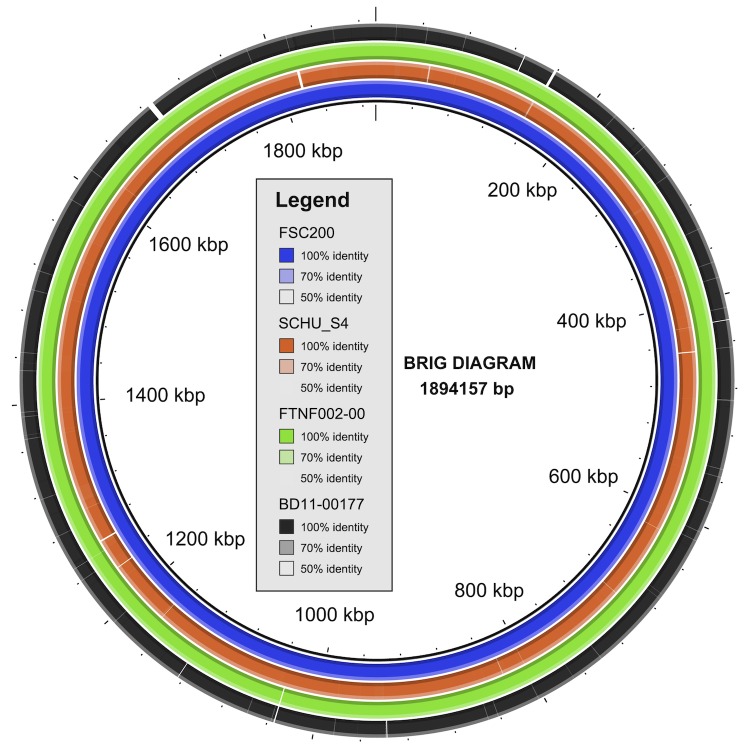
BRIG diagram of the *F. tularensis subsp. holarctica* BD11-00177, FTNF002-00 and SCHU S4 genomes using the *F. tularensis subsp. holarctica* FSC200 genome as a reference backbone. White regions represent absent genetic regions.

A BLAST Ring Image Generator (BRIG) analysis comparing the *F. tularensis subsp. holarctica* BD11-00177 genome against the *F. tularensis subsp. holarctica* genomes of F92, LVS, and FTNF002-00 revealed that the BD11-00177 draft genome shows considerable resemblance to FTNF002-00 (Figure 2).

Evolutionary history of *F. tularensis subspecies holarctica* strain BD11-00177 was inferred using publicly available whole genome sequences.

**Figure 3:**
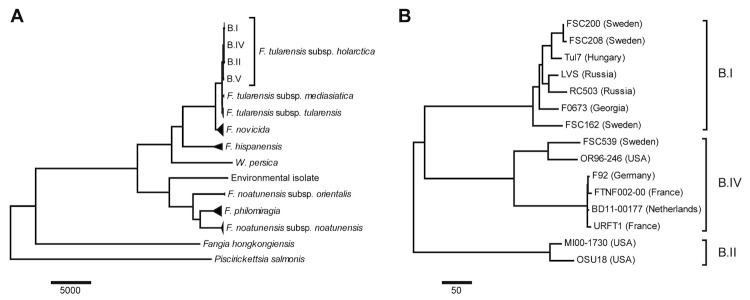
A) Overview of the *Francisella* genus phylogeny based on 52 public whole genome sequences. B) The phylogeny of *F. tularensis subsp. holarctica* strains based on whole genome sequences. The new isolate, BD11-00177 belongs to the FTNF002-00 genomic group inside the B.IV clade.

The trees in Figure 3 A and B are drawn to scale, with branch lengths in the same units as those of the evolutionary distances used to infer the phylogenetic tree. The evolutionary distances were computed using the *number of differences* method and are in the units of the number of base differences per sequence. The overview of *Francisella* genus involved 52 public genome sequences using *Piscirickettia salmonis* as outgroup (Figure 3A). The detailed analysis involved 14 *F. tularensis subsp. holarctica* genome sequences using *F. tularensis subsp. tularensis* strain SCHU S4 as outgroup (Figure 3B) [17,30,33,36-41]. All positions containing gaps and missing data were eliminated. There were a total of 1,599,589 positions in the final dataset.

## Conclusion

Here we have presented the draft genome of the first member of FTNF002-00 genomic group of *F. tularensis subspecies holarctica.* As more genetic information of members from this genomic group becomes available, a better understanding of the evolution and biogeography of this pathogen will be gained. This knowledge may help us to understand the epidemiology and potential expansion of the geographical distribution of this genomic group. Despite potential biases associated with discontinuous draft genomes, we would like to focus on the added value of draft bacterial genome sequencing. Taking advantage of low cost and high-throughput sequencing platforms allows us to probe the vast microbial diversity present in nature and rapidly respond to clinical outbreaks and acute biosecurity hazards. From an evolutionary ecology perspective, increased sequencing efforts allow us to characterize the biogeography of microbial taxa and differentiate between neutral and conserved genome contents.
